# Everybody's Page

**Published:** 1890-09-13

**Authors:** 


### Everybody's Page.

An electrical rat-trap, which instantaneously kills the victims it catches, is the latest invention from Omaha.

Three million dollars, it is said, are to be expended by a lady on a new hospital in New York for consumptives.

The British Consul at San Francisco in his last report gives some striking figures showing the magnitude of the trade in tinned, or canned, salmon and fruit. The total "pack" of salmon last year on the Pacific Coast was 1,693,800 cases, of 48 1 lb. tins each, the prime value of which was £1,812,800.

During this year a native of Galicia was sentenced to four months' imprisonment for "snatching" bodies to be ground into a powder and drunk as a charm against typhoid. Superstition dies hard in spite of the world-wide advance of medical knowledge.

"Do not take your work as a dose, and I do not think you will find it nauseous," wrote Dr. Thomas Arnold to a friend of his; "take it as a matter of course, making it your material occupation, and devote your time to it, and then you will find that it is in itself full of interest." He wrote this respecting the work of a private tutor; but it is solid advice worth taking no matter what our employment be. The reason many people fail is simply because of a want of zest about their work; to succeed one must be in earnest, and the wisdom of a man like Arnold, who set himself a great object in life and attained it so splendidly, is worth listening to.

The New York Hospital was opened for the reception of patients in 1877. The site is of limited area, and the building, which is on the street line, is carried to a height of seven stories. The neighbourhood is thickly populated. All floors are fire proof, steam elevators are placed at various points; kitchen and laundry are on the upper story, and the entire arrangements are unique. Ventilation has received special attention. The ward floors are laid with ornamental tiles, the walls are curved to the ceilings and at the ends, nickel-plated gasfittings are placed over each bed, and each patient has an electric communicator to the nurses' room. The ward furniture and fittings are ornamental as well as useful. At the rear of the centre block, on the fifth story, a tastefully arranged winter garden is constructed. Ferns, palms, and ornamental shrubs, with the addition of aquarium and cages of birds, furnish a pleasant lounge for the patients. Anyone going to New York cannot do better than to be taken ill and thus secure board and lodging in this delightful hospital.

There is a new treatment for diphtheria—viz., pine-apple juice: this is from the pharmacopœia of the Louisiana negroes!

Baking powder is not a very wholesome thing, and retards digestion, and some people are very fond of using a good deal too much of it. That made of bicarbonate of soda and cream of tartar is perhaps the easiest to digest.

The spa rooms at Buxton are to be made much larger this coming winter, the present ones being too small. We congratulate Buxton; faith in the efficacy of that health resort must be unabated.

Nothing keeps out moths so well as paper; if every good housewife when she puts away her furs pasted up all the crevices and round the lid of the box with paper, she would find her furs intact when unpacked.

Miss Marianne North, whose death has just occurred, furnishes a good example of women's work and capability nowadays. She travelled immensely, and collected botanic knowledge wherever she went, doing much for that science; some years ago she gave the Botanic Picture Gallery to Kew.

"Sister Agnes" very kindly sends us news of the successful working of some cottage homes for workhouse children; she is the sister in charge of the infirmary of the homes. She praises the wisdom of her especial Board of Guardians, who having seen how crushing life in a workhouse is to a child, built a number of handsome cottages in the midst of fields, a few miles from London where their small waifs and strays are placed. Each cottage is under the care of a "mother," who has a certain limited number of children to care for. "Sister Agnes" says she gets a trifle weary of the constant cases of ophthalmia, but the children are often such nice mites that they make up for any monotony. She tells us of a small child of three who rejoiced in the cognomen of "Fiddler," and who would often look up and say, "Fiddler loves sister." A little boy who came all disfigured with eczema, and another who, suffering greatly from a bad attack of pneumonia, would say when sister went to see him in the night, "Don't want nothing sister, do go to bed or I know you'll catch cold." Of course there are all sorts and conditions of children, some black sheep among the flock, but that the cottage homes do untold good our correspondent has ample means of ascertaining, and we shall be very glad to hear more news of "homes."

